# Molecular Pathogenesis of Central and Peripheral Nervous System Complications in Anderson–Fabry Disease

**DOI:** 10.3390/ijms25010061

**Published:** 2023-12-20

**Authors:** Antonino Tuttolomondo, Irene Baglio, Renata Riolo, Federica Todaro, Gaspare Parrinello, Salvatore Miceli, Irene Simonetta

**Affiliations:** 1 Department of Health Promotion, Maternal and Infant Care, Internal Medicine and Medical Specialties, “G. D’Alessandro”, University of Palermo, Piazza delle Cliniche n.2, 90127 Palermo, Italy; bagirene@gmail.com (I.B.); renatariolo.rr@gmail.com (R.R.); federicatodaro21@gmail.com (F.T.); gaspare.parrinello@unipa.it (G.P.); salvatore.miceli@policlinico.pa.it (S.M.); irene.simonetta@live.it (I.S.); 2 Fabry Disease Regional Reference Centre, Internal Medicine and Stroke Care Ward, University Hospital Policlinico “P. Giaccone”, 90127 Palermo, Italy

**Keywords:** Anderson–Fabry disease, galactosidase-alfa, neurological, peripheral

## Abstract

Fabry disease (FD) is a recessive monogenic disease linked to chromosome X due to more than two hundred mutations in the alfa-galactosidase A (GLA) gene. Modifications of the GLA gene may cause the progressive accumulation of globotriaosylceramide (Gb3) and its deacylated form, globotriasylsphingosine (lyso-Gb3), in lysosomes of several types of cells of the heart, kidneys, skin, eyes, peripheral and central nervous system (not clearly and fully demonstrated), and gut with different and pleiotropic clinical symptoms. Among the main symptoms are acroparesthesias and pain crisis (involving the peripheral nervous system), hypohidrosis, abdominal pain, gut motility abnormalities (involving the autonomic system), and finally, cerebrovascular ischemic events due to macrovascular involvement (TIA and stroke) and lacunar strokes and white matter abnormalities due to a small vessel disease (SVS). Gb3 lysosomal accumulation causes cytoplasmatic disruption and subsequent cell death. Additional consequences of Gb3 deposits are inflammatory processes, abnormalities of leukocyte function, and impaired trafficking of some types of immune cells, including lymphocytes, monocytes, CD8+ cells, B cells, and dendritic cells. The involvement of inflammation in AFD pathogenesis conflicts with the reported poor correlation between CRP levels as an inflammation marker and clinical scores such as the Mainz Severity Score Index (MSSI). Also, some authors have suggested an autoimmune reaction is involved in the disease’s pathogenetic mechanism after the α-galactosidase A deficiency. Some studies have reported a high degree of neuronal apoptosis inhibiting protein as a critical anti-apoptotic mediator in children with Fabry disease compared to healthy controls. Notably, this apoptotic upregulation did not change after treatment with enzymatic replacement therapy (ERT), with a further upregulation of the apoptosis-inducing factor after ERT started. Gb3-accumulation has been reported to increase the degree of oxidative stress indexes and the production of reactive oxygen species (ROS). Lipids and proteins have been reported as oxidized and not functioning. Thus, neurological complications are linked to different pathogenetic molecular mechanisms. Progressive accumulation of Gb3 represents a possible pathogenetic event of peripheral nerve involvement. In contrast, central nervous system participation in the clinical setting of cerebrovascular ischemic events seems to be due to the epitheliopathy of Anderson–Fabry disease with lacunar lesions and white matter hyperintensities (WMHs). In this review manuscript, we revised molecular mechanisms of peripheral and central neurological complications of Anderson–Fabry Disease. The management of Fabry disease may be improved by the identification of biomarkers that reflect the clinical course, severity, and progression of the disease. Intensive research on biomarkers has been conducted over the years to detect novel markers that may potentially be used in clinical practice as a screening tool, in the context of the diagnostic process and as an indicator of response to treatment. Recent proteomic or metabolomic studies are in progress, investigating plasma proteome profiles in Fabry patients: these assessments may be useful to characterize the molecular pathology of the disease, improve the diagnostic process, and monitor the response to treatment.

## 1. Background

Anderson–Fabry Disease (AFD) represents the second most prevalent glycosphingolipid storage disorder (after Gaucher disease) with a frequency of 1 in 100,000 [1]. In this disease, a deficiency of a lysosomal hydrolase, alfa-galactosidase A, causes the abnormal accumulation of uncleaved glycosphingolipids in lysosomes and other organules such as globotriosylceramide (Gb3) and its deacetylated form, globotriasylsphingosine (lyso-Gb3). AFD symptoms reflect the different organ profiles of the histopathological findings of lipid accumulation in the cardiac, renal, and peripheral and central nervous systems. Pain in Anderson–Fabry disease has been suggested as the result of degeneration of nerve fibers in the dorsal root ganglion cells with subsequent axonal degeneration of the tiny fibers involved in pain transmission patterns [2,3]. The deposition of glycosphingolipids begins in the lysosomes and causes metabolic collapse of the cells, tissue compensatory hypertrophy, cell death, and organ failure. Lipid deposits are present in the endothelium, media of small vessels, renal tubules and glomeruli, cardiac muscle, conducting fibers, and autonomic ganglia. These histopathological findings have been reported as linked to the clinical results of the disease such as renal failure, cardiomyopathy, pain crisis, and multiple cerebrovascular accidents (CVAs) [4,5]. The accumulation of globotriaosylceramide (Gb3) has been reported as the vital link between pathology and clinical symptoms in most of the involved organs. In fact, Gb3 accumulation occurs in most non-neuronal tissues and body fluids. Only central nervous system symptoms seem to be not due to a direct neuron accumulation but to the epitheliopathy of the small cerebral vessels (SVDs). The natural history of neurological Fabry patients includes transitory cerebral ischemia and strokes, even in very young persons of both genders. The pathogenetic mechanism is due to vascular endothelial accumulation of Gb-3, causing ischemic stroke or white matter lesions (WMLs) [6]. Another potential factor influencing the alteration of endothelial function is associated with Nitric Oxide Synthase-3 genotypes. Nitric oxide, produced by the endothelium, is critical in regulating vessel wall function and maintaining cardiovascular homeostasis. Also, autopsy studies in AFD [7,8] have reported the accumulation of neuronal globotriaosylceramide in specific cortical and brain stem regions, including the hippocampus. However, the clinical implications and relevance of these findings, as well as potential clinical surrogates, have not yet been explored. Despite this, the primary histological observation in AFD comprises small fiber neuropathy alongside cerebral micro- and macroangiopathy, leading to premature stroke. Cranial MRI demonstrates the presence of progressive white matter lesions (WMLs) at an early age, increased signal intensity in the pulvinar region, and the twisting and enlargement of larger blood vessels. Conventional MRI shows a gradual accumulation of white matter lesions (WMLs) resulting from cerebral vasculopathy during the progression of AFD. The peripheral neuropathy in Fabry disease causes neuropathic pain, reduced cold and warm sensation, and possibly gastrointestinal disturbances. Patients with Fabry disease suffer from pain crises from the end of the first decade of life or during puberty. In children, this pain is often associated with febrile illnesses and reduced heat and exercise tolerance. Patients describe the pain as burning, often associated with deep aches or paresthesia. Some patients also have joint pain. AFD patients may develop neuropsychiatric symptoms, such as depression and neuropsychological deficits. Due to both somatic and psychological impairment, health-related quality of life is considerably reduced in patients with Fabry disease. Targeted screening for Fabry disease among young individuals with stroke may help to reveal unrecognized cases. Furthermore, ischemic stroke is also related to inflammation and arterial stiffness, and no study has addressed this relationship in patients with AFD and cerebrovascular disease, so this topic could represent a possible future line of research [9,10]. This review aims to summarize recent developments in the understanding of molecular pathogenetic pathways involved in neurological complications of Anderson– Fabry disease. 

## 2. Molecular Pathogenesis of Anderson–Fabry Disease 

**Figure 1:**
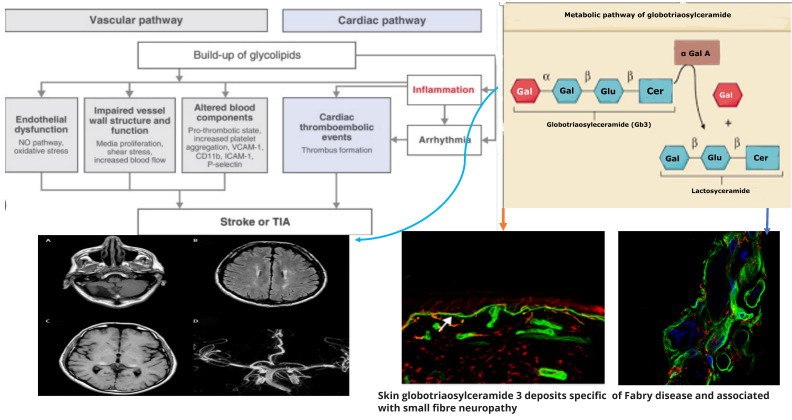
Pathogenetic mechanisms of central and peripheral nervous complications in Anderson-Fabry Disease.

In Anderson–Fabry disease (AFD), deficiency of the enzyme alfa-galactosidase A (α-GalA) leads to an abnormal buildup of globotriaosylceramide (Gb3), which is associated with end-organ damage, progressive organ failure, and subsequent clinical manifestations. An important characteristic feature of AFD is the presence of distinct lipid deposits known as “zebra bodies”, which are prominently observed in various cell types, particularly at endothelial levels [11]. Previous studies have identified the main constituent of these abnormal deposits as globoside globotriaosylceramide (Gb3), previously referred to as ceramidetrihexoside (CTH) [11]. Moreover, other abnormal glycosphingolipids such as galactosylceramide (Gb2) and blood group B, B1, and P1 antigens, sharing a terminal α-galactosyl moiety, have been described in end-organ damage of AFD patients [3]. The molecular pathogenesis of this lipid disorder has been investigated in a prior study, suggesting a potential causal relationship between the deficiency of lysosomal acid alfa-galactosidase activity and the impaired conversion of Gb3 to lactosylceramide (LacCer) [12]. Notably, α-GalA has been shown to play a crucial role in the degradation of the intermediate metabolite globoside Gb3, and the involvement of α-GalB in the metabolism of this metabolite has been reported in several studies [7,13]. The α-GalA enzyme is derived from a precursor consisting of 429 amino acids, which transforms to form a homodimer with 398 amino acids [8,14]. The three N-linked glycans of α-GalA receive mannose-6-phosphate moieties collaborating with the enzyme’s arrival to lysosomes by mannose-6-phosphate receptors. The activity of α-GalA toward the lipid substrate is increased by the activator protein saposin B and negatively charged lipids [3]. Over 1000 mutations have been identified in the GLA gene, primarily consisting of missense mutations. However, the complete pathogenic implications of several of these mutations remain unclear. Some α-GalA mutations do not appear to be associated with reduced α-galactosidase activity, leading to uncertainty about their actual pathogenic role. Over and above the cell Gb3-deposits, the end-organ damage in AFD may also be related to the immunoinflammatory mechanism (see Figure 1). Nevertheless, it has been reported that the accumulation of Gb3 [7,8,14] could be associated with specific molecular mechanisms, and early intervention through therapy may prevent the progression of organ failure. Valbuena et al. indicated a crucial pathogenetic role of the overloading of lysosomes with Gb3 and subsequent damage of cytoplasm and subsequent cell death [13]. Additionally, the Gb3 deposits and the resulting organ damage may also be influenced by inflammatory processes [7]. Recently, Gb3 has been reported as potentially identifiable as CD77 [8], which has a direct effect on apoptosis and necrosis [13]. Furthermore, according to Rozenfeld et al., individuals with Anderson–Fabry disease (AFD) exhibit disturbances in leukocyte function when compared to the progressive involvement of other immunocompetent cells, including lymphocytes, monocytes, CD8+ cells, B cells, and dendritic cells [14]. However, another study found no correlation between inflammatory biomarkers such as C-reactive protein and the Mainz Severity Score Index (MSSI), an index used to assess the clinical severity of AFD [15]. Some authors have suggested that there is an immune response against the enzyme, leading to alfa-galactosidase A deficiency. Moore et al. reported a higher degree of neuronal apoptosis due to the neutralization of anti-apoptotic molecules in pediatric AFD [16]. Finally, some authors indicated that Gb3 accumulation may enhance oxidative stress and the production of reactive oxygen species (ROS) [17]. Another interesting point of endothelial function may be due to the Nitric-Oxide-Synthase-3-genotypes. Endothelium-derived nitric oxide plays a crucial role in regulating vessel dilation and maintaining vascular homeostasis. There is a genetic variant of the Nitric-Oxide-Synthase-3 (NOS3) gene that has been identified as a potential factor in disrupting this homeostasis, and it appears to be associated with a reduced thickness of the posterior wall of the left ventricle [18]. This finding may offer insights into the pathogenesis of various cardiac phenotypes observed in Fabry disease. Additionally, Wang et al. reported the presence of Gb3 storage in pulmonary smooth muscle cells and the vascular endothelium of a female patient with Anderson–Fabry disease [19]. This observation further supports the involvement of Gb3 accumulation in the disease’s pathogenesis, particularly in vessels. Deacylated globotriaosylceramide (lyso-globotriaosylceramide, lyso-Gb3) has been reported as increased in patients with AFD. Lyso-Gb3 abnormal deposits cause hypertrophy of smooth muscle cells in vitro and hyperplasia of the internal layer of arterioles [20]. The pathogenesis of Anderson–Fabry disease (AFD) involves various cell types, including endothelial and smooth muscle cells, cardiac cells at the myocardial and valvular level, tubular and glomerular cells, as well as podocytes and peripheral nervous cells [21]. Cerebrovascular involvement, particularly affecting perforating arterioles, is a determining factor in morbidity and mortality in individuals with AFD. The pathophysiological mechanisms underlying end-organ damage in AFD are intricate and challenging to describe due to their complexity. The initial clinical manifestations of cerebral AFD primarily involve the microvasculature. As indicated in the aging process, arterial remodeling and intima-media thickening in medium-to-large caliber vessels have been reported as an important step in cerebrovascular complications of AFD [22].

Neurological symptoms in patients with Anderson–Fabry disease (AFD) can be attributed to the involvement of both large and small vessels within the central nervous system. Ischemic cerebral events resulting from large artery involvement may arise from thrombosis of the major intracranial vessels, cardioembolic mechanisms, or possibly atherothrombotic events [23]. In addition to the aforementioned mechanisms, another potential pathogenic factor contributing to cerebrovascular complications in AFD is the presence of distinctive small vessel disease. This implies that multiple pathways and vessel sizes are implicated in the development of neurological symptoms in AFD patients. The clinical pathways of cerebrovascular complications in AFD seem to be the small vessel disease and some neuroimaging patterns appearing as either subcortical stroke or the frequently asymptomatic white matter lesions (WMLs) and subcortical infarcts [23,24,25]. In patients with AFD, strokes occur in both the anterior and the posterior circulatory systems, as well as in cortical and subcortical locations. However, the mechanism and topography of such strokes represent not fully clarified issues because of the paucity of studies addressing this issue in all stroke subtypes as opposed to most studies of cryptogenic stroke [26,27].

## 3. Molecular Pathogenesis of Central Nervous System Involvement in Anderson–Fabry Disease

**Figure 2:**
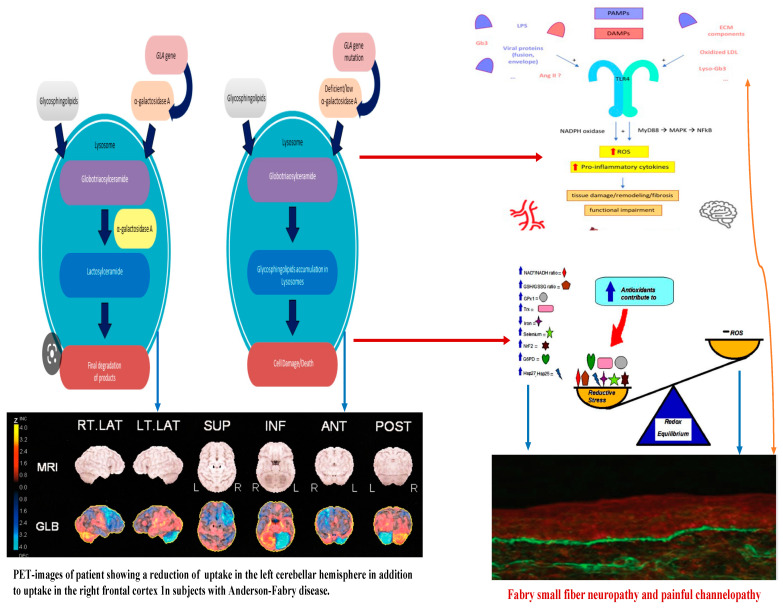
Linkage between metabolic pathogenesis of Anderson-Fabry disease and inflammatory and oxido-reductive pathogenesis of Gb3-related organ damage.

Neurological symptoms associated with Fabry disease encompass both peripheral nervous system and central nervous system involvement. In AFD, peripheral nervous system involvement manifests as severe neuropathic pain, as depicted in Figure 1 and Figure 2. Additionally, other non-central nervous clinical manifestations include autonomic dysfunction, characterized by symptoms such as reduced sweating (hypohidrosis), abdominal pain, intestinal dysmotility disorders, and arrhythmias [28,29]. Autoptic studies revealed Gb3 storage in autonomic ganglions using immunohistochemical staining [30]. At skin biopsy, some studies reported how Fabry patients are characterized by a progressive reduction in intra-epidermal innervation associated with small fiber sensory neuropathy [31]. Although the pathogenetic basis of peripheral neuronal pathology in Fabry disease is understood, mechanisms involving central neurons remain incompletely elucidated. While Gb3 deposits have been observed in ganglion locations, explaining the autonomic and peripheral nervous system involvement [32], no definitive evidence of Gb3 deposits in central neurons has been reported. However, lyso-Gb3 levels are increased in the plasma and tissues of experimental rat models with AFD and the plasma of male subjects with classical pathogenetic mutations [13]. The elevated levels of lyso-Gb3 are a potential pathogenic factor contributing to the pathology of AFD. In fact, lyso-Gb3 plays a role in the painful damage associated with deposits in dorsal root ganglia neurons. Further investigation is needed to understand the pathogenic mechanisms underlying central neuronal involvement in Fabry disease. Recently, Choi et al. reported how the administration of lyso-Gb3 caused a high degree of stimulation of pain-transmitting neurons of normal mice and that lyso-Gb3 caused an increase in Ca2 + influx in not-AFD root ganglion cells cultured from adult mice [33]. Furthermore, in other lysosomal storage disorders, such as Gaucher disease, some authors reported how glucosyl sphingosine (glucopsychosine), an analogue of lyso-Gb3, has a toxic effect on cultured neuronal cells [34]. Patients with Anderson–Fabry disease (AFD) are susceptible to cerebrovascular disease, and this condition is observed more frequently in young individuals [34,35,36]. They may experience ischemic strokes at a higher rate compared to the general population of similar age groups. Furthermore, neuroimaging studies often reveal findings indicative of chronic cerebrovascular disease, which can subsequently lead to cognitive impairment [37,38]. This underscores the importance of monitoring and managing cerebrovascular complications in AFD patients to mitigate the risk of stroke and cognitive decline. Other neurological AFD symptoms due to peripheral and autonomic nervous involvement, such as typical pain, sensory disturbances, and hypohidrosis, have been reported. MRIs of the brain reported ischemic lesions with a higher frequency of cerebellum and brainstem localizations regardless of the presence of neurological signs, whereas T2 MRIs often reveal white matter lesions (WMLs) with hyperintensities which, according to some authors, resemble MRI brain findings characteristic of demyelinating diseases. Some studies have reported that sensory nerves from patients with AFD show several morphological and functional abnormalities [38,39,40], such as lower myelinated and unmyelinated fiber presence, lipid deposits in various cell types, myelin abnormality, and disorders of glial cells. Previous studies reported Gb3 accumulation and swelling of dorsal root ganglia (DRG) neurons in patients [39] and AFD rodent models (38). However, the extent of Gb3 accumulation or other pathologies of peripheral nerves in FD remains unclear. AFD rodent models are a powerful tool for the characterization of nerve pathology. If animal models reproduce human pathology, they could be utilized to understand pain mechanisms for patients with AFD [40,41,42]. In a study [43], the authors reported neuronal damage development of an in vitro model system with a useful model of neuronal functional disturbance in Fabry disease by using short-hairpin RNA to create a stable knock-down of AGA in the human cholinergic neuronal cell line, LA-N-2. The authors reported that these knock-down cellular lines show low levels of AGA activity and Gb3 accumulation. Furthermore, in experimental knock-out cells, the release of neurotransmitter acetylcholine appears to be significantly reduced. This confirms that this experimental model is adequate as a neuronal function model with a disturbance of neurotransmitter release possibly characteristic of AFD. The neuronal pathway involved in the pathogenesis of pain crisis and neuropathic disturbances in AFD is not due to the involvement of peripheral structures such as dorsal root ganglia (DRGs) as well as other nuclear regions of the CNS. In reverse, spinal and supraspinal nuclei and cerebral areas are involved in pain transmission and the anterior cingulate cortex in AFD [41,42]. Gb3 accumulation was documented also in the central nervous system, particularly in the hippocampus and cortical layers [40], further confirmed in the AFD mouse model [41,42]. Moreover, abnormalities of gene expression in AFD have been reported in crucial brain regions that have an important role in the development of the AFD pain pathologic phenotypes, such as prefrontal and sensory cortices, insular cortex, and basal ganglia circuits. These are all regions that have a direct role in the procession of abnormal pain signals involved in the pathogenesis of chronic neuropathic pain, and maybe also in AFD [40]. Cerebrovascular ischemic events in AFD are the result of cerebral microvessel occlusion, which is associated with progressive wall thickening caused by the accumulation of glycolipids, leading to both thrombotic and non-thrombotic lumen occlusion. Some researchers [44] have reported an abnormality in Gb3 metabolism within central nervous system (CNS) neurons. Additionally, these authors have observed that in AFD, neuronal swelling is likely due to disturbances linked to Gb3 accumulation, particularly in specific nuclei such as the amygdaloid body, the subiculum, and the dorsal vagus nucleus of the medulla oblongata [44]. These findings further support the notion that the pathogenesis of globotriaosylceramide deposits in AFD is not yet fully understood [44].

Concerning the pathology of cerebral vessels in AFD, the involvement has been observed [45,46,47,48] of the subarachnoidal arteries of medium size with narrowing of the lumen due to intimal fibrosis mixed with smooth muscle cells (SMC), with membrane abnormalities and stiffening of internal elastic tunica due to the total or partial change of the medial SMC and subsequent fibrosis and adventitial fibrosis. AFD involves smooth muscle too, but it is not clear whether the first step in Fabry vasculopathy involves endothelial cells, with a subsequent prothrombotic state, or if it begins in the smooth muscle cells of the arterial media layer [45,46,47,48]. It has been reported that lyso-Gb3 plays a crucial role in the pathogenesis of Fabry vasculopathy, and it has been proposed that smooth muscle cells, rather than endothelial cells, represent the main target of cell accumulation. Smooth muscle cells exposed to lyso-Gb3 proliferate, and this proliferation has been reported as linked with the hypertrophy of arterial walls [46,47,48,49,50]. Accumulation of lyso-Gb3 within the media layer of the arteries may also promote cell proliferation, with the fibrotic remodeling of the arterial wall leading to arterial wall stiffness. Shear stress has also an important role in increasing the degree of exposition of angiotensin 1 and 2 receptors in endothelial cells; enhancing the production of reactive oxygen species, NF-κB, β-integrin, and cyclooxygenase 1 and 2 activity; and lowering nitric oxide synthesis (43). All these cellular and biochemical events seem to represent the candidate step to initiate an inflammatory cascade with prothrombotic and pro-inflammatory effects on leukocytes, endothelial cells, and vascular smooth muscle cells [47]. Indeed, inflammatory pathogenesis of Anderson–Fabry disease (AFD) complications in the central nervous system (CNS) has been previously documented [48]. It is important to note that the CNS serves as the primary target for numerous lysosomal storage disorders. In many lysosomal genetic diseases, inflammation mechanisms in the CNS involve microglial cells and astrocytes. Lysosomes, after cellular damage, produce Pathogen Associated Molecular Patterns (PAMPs) or Damage Associated Molecular Patterns (DAMPs) by the astrocytes and microglial cells, using Toll-like receptors T that enhance the cytokine release causing inflammation and cellular death. Ischemic stroke is considered one of the most extensively discussed potential complications of central nervous system (CNS) involvement in Fabry disease. Its pathogenesis involves inflammatory or degenerative occlusive processes affecting the arterial wall or (micro) embolic mechanisms. In the context of stroke pathogenesis, three vascular events related to cerebrovascular structures are influenced by inflammation. These events encompass endothelial cell dysfunction, impaired vessel wall structure and function, as well as alterations in blood components. Indeed, the progressive accumulation of Gb3 in the endothelial cells of intracranial blood vessels has been identified as the primary vasculopathy event associated with ischemic stroke pathogenesis in AFD [22]. Besides Gb3 accumulation, other pathogenetic factors may contribute to the development of ischemic stroke in AFD patients. These factors include possible acquired thrombophilic conditions, abnormalities in intravascular flow velocity (either impaired or increased), autonomic dysfunction [51], and oxidoreductive damage [52]. All these factors collectively play a role in the complex pathogenesis of ischemic strokes in individuals affected by AFD. The role of vascular or autonomic dysfunction as a pathogenic mechanism has been reported as able to impair cerebral blood flow velocities and cerebral autoregulation [51]. In their study, some authors [51] assessed transcranial Doppler sonography in Fabry patients and examined various parameters, including the resistance index, pulsatility index, cerebrovascular resistance, spectral powers of oscillations in RR intervals, mean blood pressure, and mean cerebral blood flow velocities. Their findings indicated a reduction in blood flow velocity, which was attributed to the involvement of certain branches of the middle cerebral artery caused by reduced sympathetic tone and/or progressive arterial stiffening. Additionally, abnormal blood flow oscillations were observed to impair the autoregulation of blood pressure directed to the brain. These observations highlight the potential impact of cerebrovascular changes on the pathophysiology of Fabry disease and its effects on cerebral blood flow dynamics. Thus, both reduced cerebral blood flow velocities and impaired cerebral autoregulation are likely to be involved in the increased risk of cerebrovascular complications in AFD.

## 4. Molecular Pathogenesis of Peripheral Nerve Involvement in Anderson–Fabry Disease

Neuropathic pain represents a significant clinical aspect of Anderson–Fabry disease [49,50]. Patients with AFD often experience pain in their hands and feet, along with severe episodic pain attacks known as ‘Fabry crises’. This pain is associated with the accumulation of Gb3 in pain-sensitive neurons of the dorsal root ganglia (DRG). The abnormal transmission of pain signals is linked to disturbances in ion channel function [50]. Among these channels, acid-sensing ion channels (ASIC) have been studied in connection with pain [53,54]. In the central nervous system (CNS), ASICs are located in areas highly involved in pain perception. Hyperalgesia, or increased sensitivity to pain, in AFD is attributed to an upregulation of ASIC activity, as observed in animal models and AFD patients [44]. ASIC channels act as proton sensors in the nervous system and play a crucial role in pain transmission. Furthermore, in AFD, elevated levels of Gb3 and lyso-Gb3 have been linked to chronic pain, and this association seems to be closely related to Trpv channels, and potassium, calcium, and sodium channels, which have been extensively studied in DRGs of AFD animal models [55]. In addition, some authors [56] analyzed the pathological nerve findings in AFD rat models [57,58]. They observed a pathological breakdown of Gb3 in lysosomes in AFD, and they correlated peripheral nerve pathology with the accumulation of Gb3 or lysosomes in the axons [57,58]. Morphological abnormalities in peripheral nerves have also been reported in patients with FD [57,58]. These findings shed light on the complex mechanisms underlying the neuropathic pain experienced by individuals with AFD and offer insights into potential targets for therapeutic interventions. Authors [59] who studied the saphenous nerve (sensory), the tibial nerve (mixed sensory/motor) at proximal and distal locations, and the femoral motor branch, reported a significant decrease in myelinated fiber frequency in the saphenous (sensory) and distal tibial nerves (mixed sensory/motor) of AFD rats. Also, a low degree of intra-epidermal nerve fiber density nerve fiber density (IENFD) in patients with AFD [59,60] has been reported. No abnormality in myelinated fiber density has been reported in AFD proximal tibial (mixed sensory/motor) or femoral motor branches [59,60], whereas anatomical abnormality of unmyelinated fiber has been observed in the tibial nerve (mixed sensory/motor) [61]. Authors have further shown abnormality in the density of unmyelinated fibers in the saphenous (sensory) and femoral motor branch nerves and a lower frequency of unmyelinated fiber density in the saphenous (sensory) but not femoral motor branch of AFD nerves. Indeed, a characteristic osmophilic accumulation in myelinated axons of the proximal tibial nerve has been reported [61]. In AFD, some abnormalities concerning myelinated Aδ fiber conduction have been reported [61]. In rat models of AFD, 25% of myelinated axons showed significant lipid accumulation that may represent the pathogenetic explanation of myelinated Aδ fiber dysfunction observed in patients with AFD [59,60,61]. C-fiber dysfunction has also been reported in patients with AFD with subsequent abnormalities in pain thresholds and heat and cold sensitivity [58]. The finding concerning the axon’s diameter in abnormal function of unmyelinated fibers seems to be indicative of altered conduction in the C fibers of AFD peripheral nerves, and this finding is one of the pathogenetic bases of the AFD-characteristic pain crisis. Small sensory nerves, myelinated Aδ fibers, play a main role in transmitting mechanical pain sensitivity, such as unmyelinated C fibers working with warm sensations and pain sensitivity to heat. In AFD, small fiber disease involves Aδ fibers [62,63,64]. Thermal sensation abnormality has been reported as mainly affecting the feet more than the hands with a progressive proximal sequential involvement. The first thermal abnormality involves cold perception (Aδ fibers) more than warmth sensitivity (C fibers), [65] indicating how the thinly myelinated Aδ fibers seem to be more prone to be involved in the Gb3 accumulation peripheral nerve damage [66]. Autonomic involvement in AFD has been described as the cause of gastrointestinal dysmotility (e.g., abdominal cramps, bloating, diarrhea, and nausea), hypohidrosis, abnormality of pupillary constriction, impaired tear and saliva formation, Raynaud phenomena, cardiac rhythm disturbances, and orthostatic hypotension [67,68]. Autonomic dysfunction also regards sudomotor nerve fibers and sweat gland function that have been reported as affected in AFD patients without treatment [69]. Sural nerve bioptic samples showed a characteristic reduction in small myelinated and unmyelinated nerve fibers [70,71]. Glycolipid deposits have been reported in the perineurium, sensory ganglia, vascular smooth muscle cells (SMCs), fibroblasts, and endothelial cells [69]. Additional bioptic studies have revealed a significant decline in nerve fibers as individuals age, systemic compromise, and kidney involvement [69]. The first pathogenetic hypothesis is based on the presence of Gb3 deposits in dorsal root ganglion (DRG) neurons driving neuronal damage with a subsequent ganglionopathy resulting in reduced intra-epidermal nerve fiber density (IENFD) [63,72,73]. Gb3 accumulation affects and impairs the function of cellular membrane proteins, such as ion channels, with subsequent abnormalities of excitability leading to cytotoxicity and nervous fiber dysfunction and damage. This hypothesis fits well with the reduction in intra-epidermal nerve fibers in patients with AFD, also found in the skin on the back, which is normally preserved from intra-epidermal fiber loss in length-dependent peripheral neuropathies [34]. Another pathogenetic hypothesis is microangiopathy of the vasa nervorum due to an ischemic mechanism caused by Gb3 deposition within the endothelial cells of the blood vessels [74,75]. Also, according to the literature, lyso-Gb3 seems to be a stimulus to SMC proliferation in vitro, and it is involved in the development of vascular pathology in AFD [75]. Finally, a plausible hypothesis is linked to an aberration in excitation and signal transmission of neurites in pain-transmitting neurons due to myelin abnormalities [74,75] caused by nerve fiber reduction.

## 5. Conclusions

Glycosphingolipid deposits in endothelial and smooth muscle cells and neurons of the autonomic nervous system are the main pathogenetic mechanism of Anderson–Fabry disease (AFD). Microvascular complications (such as brain disease) are clinical symptoms of the central nervous system in AFD, with no well-understood pathophysiology. Some studies indicate that vascular lesions of Anderson–Fabry disease may be related to endothelial dysfunction, changes in cerebral perfusion, and prothrombotic state [59,60,61]. To date, a not fully resolved question is the issue concerning the role of accumulation of Gb3 in endothelial cells and the role of smooth muscle cell proliferation in the medial arterial layer as the real first pathogenetic event of Anderson–Fabry vascular disease. Fiber neuropathy seems to impair vascular reactivity, whereas accelerated atherogenesis has been described in patients with AFD and various degrees of organ damage. Some studies have shown a high thickness of the intima-media of different arterial sites [59,60]. Cerebrovascular disease can progress asymptomatically in the early stages of Fabry disease, as indicated by a recent study [9,52,76] employing transcranial Doppler (FTC) examination. Authors reported that AFD subjects showed reduced resting blood velocity, underlying a disturbance of neurovascular coupling in the visual cortex. These findings indicate how patients with AFD may develop vascular dysfunction in the posterior circulation territory early in the natural history of the disease. The neurological complications of Fabry disease have for many years been loosely attributed to Gb3 accumulation. However, how much neurological damage results from the accumulation of toxic metabolites is strictly dependent on the central or peripheral nature of the damage. The accumulation of Gb3 seems, in fact, to have a more evident role at the peripheral level with the clinical epiphenomena represented by the “pain crisis” and by the skin biopsy finding of the subcutaneous and periglandular peripheral nerve rarefaction. At the level of the central nervous system, the role of accumulation appears to be substantially less ambiguous, with a proven pathogenic role of cerebral arteriolopathy due to the characteristic epitheliopathy of Fabry disease. The characterization of the role of pathogenic mechanisms alternative to accumulation in the neurological complications of AFD could offer in the future other therapeutic targets for enzymatic replacement or stabilization, such as, for example, the modulation of neuroinflammation, cerebral arteriolar remodeling therapy, and the control of candidate pathogenetic oxidoreductive distress markers. Nevertheless AFD patients progressively develop further cerebrovascular risk due to metabolic and inflammatory pathogenetic mechanisms linked to classical stroke risk factors such as age, hypertension, diabetes, and dyslipidemia [77,78,79,80,81,82,83,84,85,86,87,88,89].
